# *Holothuria* (*Mertensiothuria*) *viridiaurantia* sp. nov. (Holothuriida, Holothuriidae), a new sea cucumber from the Eastern Pacific Ocean revealed by morphology and DNA barcoding

**DOI:** 10.3897/zookeys.893.36013

**Published:** 2019-12-02

**Authors:** Giomar Helena Borrero-Pérez, María Juliana Vanegas-González

**Affiliations:** 1 Instituto de Investigaciones Marinas y Costeras – INVEMAR, Museo de Historia Natural Marina de Colombia (MHNMC) - Makuriwa, Calle 25 No. 2–55, Playa Salguero, Rodadero, Santa Marta, Colombia Museo de Historia Natural Marina de Colombia Santa Marta Colombia

**Keywords:** 16S, COI, Chocó, Colombia, Echinodermata, Cabo Marzo, Gulf of Cupica, Gulf of Tribugá, Holothuroidea, mtDNA, morros, riscales, rocky reefs

## Abstract

Holothuria (Mertensiothuria) viridiaurantia**sp. nov.** is described based on specimens from rocky reefs of northern Chocó in the Colombian Pacific Ocean; however, it also occurs along the Eastern Pacific Ocean from Mexico and Panama. Although specimens from Mexico and Panama were previously identified as Holothuria (Mertensiothuria) hilla Lesson, 1830 the new species is easily distinguished morphologically and via mtDNA. In terms of morphology, the species can be identified by its olive-green background and white-orange papillae and tentacles, larger tentacles with deep indentations and also by larger buttons on the dorsal and ventral body wall, papillae and tube feet; large, thick and rough tentacle rods, and the absence of ossicles in the longitudinal muscles. The new species is included in the subgenus Mertensiothuria considering molecular evidence.

## Introduction

The family Holothuriidae Ludwig, 1894 currently includes 211 valid species, with the genus *Holothuria* Linnaeus, 1867 being the most diverse, containing 165 formally described species (WoRMS 2019a). Sixteen new *Holothuria* species have been described from different localities around the world since 2000; two of them from the Central and Tropical Eastern Pacific (Laguarda-Figueras and Solís-Marín 2009; Honey-Escandón et al. 2011). The diversity of *Holothuria* will likely continue to grow considering some “cryptic” species currently recognised based on molecular evidence (COI mtDNA) and morphological characteristics, such as colouration, as reported for the Holothuria (Thymiosycia) impatiens complex (Michonneau 2015). In addition, the exploration of poorly known regions could generate new information on the diversity of holothurians and other marine organisms. In particular, an area that warrants further exploration is the north of the Colombian Pacific (Chocó), part of the Tumbes-Magdalena-Chocó biogeographical hotspot that is considered a mega-diverse area (Cortés 1997).

Holothuria (Mertensiothuria) Deichmann, 1958, one of the 18 *Holothuria* subgenera, was reviewed by Samyn and Massin (2003), who emended its diagnosis by incorporating ossicles from the longitudinal muscles. According to Samyn and Massin (2003) there were six species in the subgenus; four of them were previously recognised: H. (M.) albofusca Cherbonnier, 1988, H. (M.) fuscorubra Théel, 1886, H. (M.) leucospilota (Brandt, 1835), and H. (M.) papillifera Heding in Mortensen, 1938; and two were transferred from the subgenus Thymiosycia into *Mertensiothuria*: *H. hilla* Lesson, 1830 and *H. aphanes* Lampert, 1885. Samyn and Massin (2003) also removed four species from the subgenus either because of the absence of ossicles in the longitudinal muscles (*Holothuria arenacava*Samyn et al., 2001 and *Holothuria platei* Ludwig, 1898) or because this characteristic was unknown (*Holothuria artensis* Cherbonnier & Féral, 1984 and *Holothuria exilis* Koehler & Vaney, 1908). According to WoRMS (2019b), *Mertensiothuria* currently includes the same six species accepted by Samyn and Massin (2003), although H. (M.) papillifera is considered species inquirenda. In addition, the subgenus includes three more species: H. (M.) isuga Mitsukuri, 1912, H. (M.) arenacava and H. (M.) artensis; the last two species excluded in the revision by Samyn and Massin (2003) have been transferred by WoRMS but with no reference.

Among the species in this subgenus H. (M.) hilla Lesson, 1830 is the most widespread species, reported from the Red Sea to Madagascar and across the Indian Ocean and the Pacific Ocean to the Central and Tropical Eastern Pacific (Purcell et al. 2012). It is a common species in the Central and Tropical Eastern Pacific occurring in its common colour morph, comprised of a yellow background and white papillae (Samyn and Massin 2003; Purcell et al. 2012). However, specimens with different colours, such as an olive-green background and white-orange papillae, have been reported by several authors (Solís-Marín et al. 2009, Lam. 30A; Sotelo-Casas et al. 2015: fig. 2E; Molina et al. 2015: fig. 3C). Specimens with yellow and green colour patterns were collected in the Colombian Pacific Ocean in 2016, allowing comparison of the morphology and mitochondrial DNA. The purpose of this paper is to describe a new species of *Holothuria* from the Eastern Pacific and to indicate how it differs from Holothuria (Mertensiothuria) hilla.

## Materials and methods

**Figure 1.**
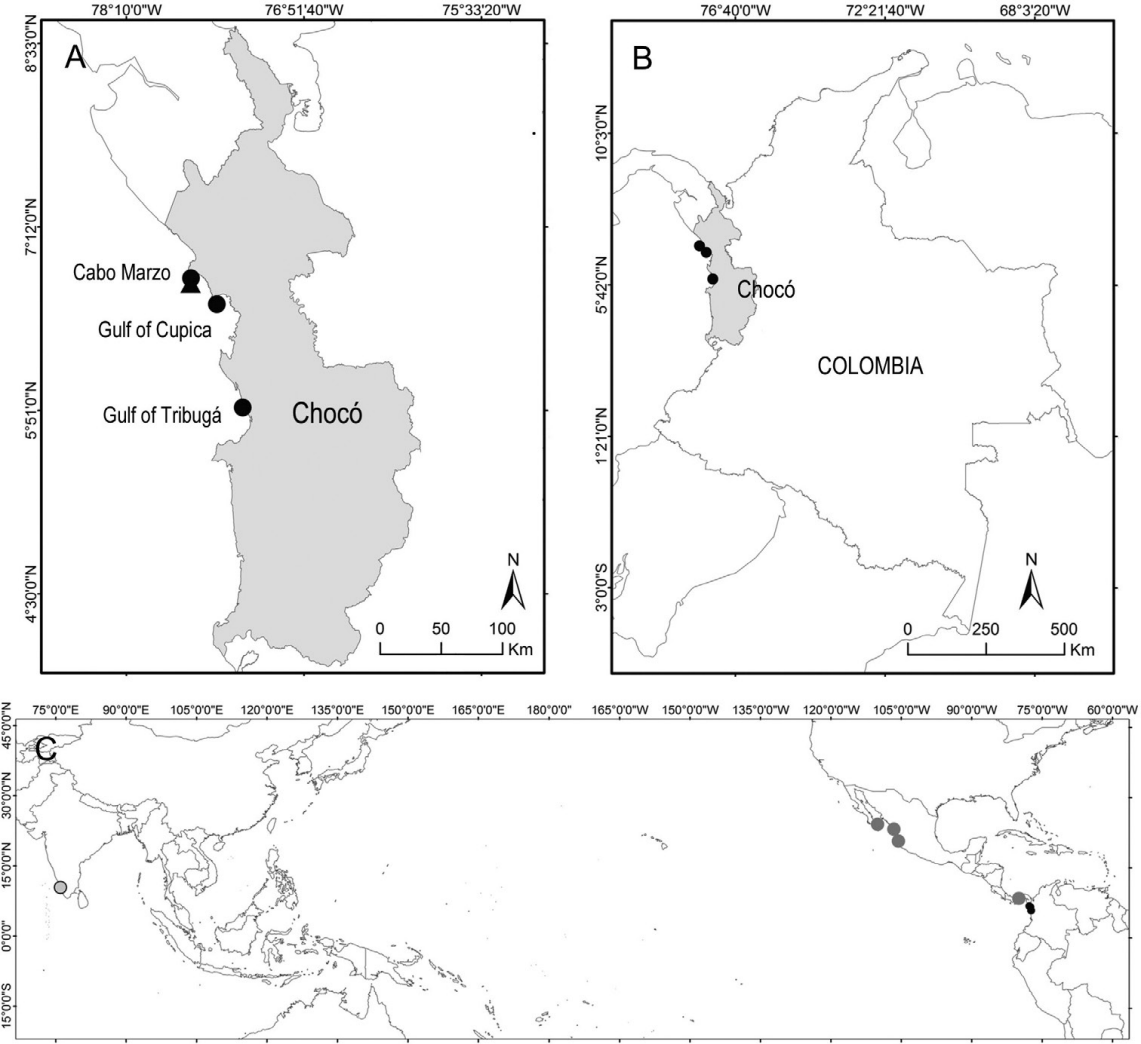
Maps showing the geographic distribution of Holothuria (Mertensiothuria) viridiaurantia sp. nov. **A, B** Detailed distribution in Chocó, Colombia; the triangle indicates the only locality were H. (Mertensiothuria) hilla specimens were collected **C** Wider distribution of H. (M.) viridiaurantia sp. nov. showing other localities from Panama and Mexico where the species have been identified through photographs, and the locality in India from where the GenBank sequence KP780302 originated. Colombian localities are represented by exact coordinates, and all other localities were derived from specific localities mentioned in the literature.

The specimens reviewed were collected as part of the project “Riscales”, developed by the Instituto de Investigaciones Marinas y Costeras – INVEMAR (www.invemar.org.co), seeking to characterise the biodiversity of the rocky reefs (called locally “riscales” and “morros”) located in northern Chocó in the Colombian Pacific Ocean. These ecosystems are important for regional fisheries and conservation (Díaz-Fahrenberger et al. 2016). Specimens were collected by hand using SCUBA diving at three rocky reefs between 10 and 15 m depth, during two sampling events in April and October 2016 (Fig. 1A, B). The specimens were placed in plastic bags with seawater, relaxed using magnesium chloride, fixed and preserved in 96% ethanol. They are deposited at the Museo de Historia Natural Marina de Colombia (MHNMC) – Makuriwa of INVEMAR **(INV EQU).**

External and internal morphology were reviewed to record standard data for sea cucumbers. Tissue from papillae, dorsal body wall, tube feet, ventral body wall, tentacles, and internal organs (longitudinal muscles, respiratory trees, tentacle ampullae, cloaca, and intestine) was removed and dissolved in fresh household bleach. Ossicles were observed and photographed using light microscopy and, at least, ten ossicles of each type were measured using the software ImageJ (Schneider et al. 2012). Type of ossicles, shape, and size were compared with those described by Lesson (1830) and Samyn and Massin (2003).

Ethanol-fixed tissues of the sea cucumbers collected during the project were processed to obtain sequences of the mitochondrial cytochrome oxidase I (COI) and 16S (large subunit) genes; in this paper, only the data of the specimens of interest are shown. Genomic DNA was extracted using the QIAGEN extraction kit (DNeasy Blood & Tissue Kit) and COI and 16S were amplified using the primers COIceF (ACTGCCCACGCCCTAGTAATGATATTTTTTATGGTNATGCC) and COIceR (TCGTGTGTCTACGTCCATTCCTACTGTRAACATRTG) (Hoareau and Boissin 2010) and 16SA (CGCCTGTTTATCAAAAACAT) and 16SB (CTCCGGTTTGAACTCAGATCA) (Palumbi 1996). PCRs were carried out following the conditions described by Hoareau and Boissin (2010). PCR products were purified and sequenced using the BigDye 3.1 (Applied Biosystems) technology. The obtained nucleotide sequences were edited using Mega 7. We analysed a fragment of 443 bp of 16S genes (including gaps) and 439 bp of COI. Sequences of COI were translated into amino acids to ensure their integrity and accuracy. The sequences obtained in the present study were submitted to GenBank (Table 1). Available sequences of H. (M.) hilla, H. (M.) leucospilota, H. (T.) arenicola, and H. (T.) impatiens from GenBank were included in the analysis (Table 1). 16S sequences were aligned using the L-INS-i method implemented in MAFFT 6 (Katoh et al. 2002) and COI with Clustal W (Thompson et al. 1994). Distances using Kimura 2 parameters correction were calculated and neighbour-joining trees were generate using Mega 7 (Kumar et al. 2016). Within-group genetic differences were analysed on the species level also based on Kimura 2- parameter distances. The best substitution model was searched using the Akaike information criterion implemented in jMoldelTest (Posada 2008). Phylogenetic relationships were inferred using Bayesian Inference (BI) and Maximum Likelihood (ML). BI was performed with MrBayes v. 3.2.6 (Ronquist and Huelsenbeck 2003) using unlinked GTR+G evolutionary model for each gen; the data set was run twice, using four Markov chains for ten million generations for each analysis to estimate posterior probabilities. ML analysis was performed in Mega 7; support was assessed in this case by 1000 bootstrap pseudoreplicates.

**Table 1.** Specimens of Holothuria (Mertensiothuria) viridiaurantia sp. nov. from Colombia and GenBank sequences analysed for the partial cytochrome oxidase subunit 1 (COI) and/or 16S genes. Sequence-Voucher Location column includes the origin of the sequences according to the GenBank references where they have been generated; sequences from this study include the catalogue number at the MHNMC-INVEMAR (INV EQU). An asterisk (*) in the species column indicates changes in the GenBank ID, where the sequences were previously identified as H. (Mertensiothuria) hilla.

Species	GenBank Accession number		Sequence-Voucher, Location	Reference
	COI	16S		
H. (M.) viridiaurantia sp. nov.	MK477997	MK477991	Colombia (Pacific) Holotype (INV EQU4309)	**This study**
	MK477998	–	Colombia (Pacific) Paratype (INV EQU4312)	**This study**
	–	MK477992	Colombia (Pacific) Paratype (INV EQU4234)	**This study**
H. (M.) viridiaurantia sp. nov.*	JN207616	JN207515	Mexico	Honey-Escandón et al. 2012
	KP780302	–	India	Deepa and Bijukumar, unp.
H. (M.) hilla	MK477996	MK477994	Colombia (Pacific) (INV EQU4311)	This study
	–	MK477993	Colombia (Pacific) (INV EQU4310)	This study
	KX874337	KX856783	Mariana Islands, Guam	Miller et al. 2017
	–	EU822442	–	Uthicke and Byrne, unp.
	–	FJ223856	Malaysia	Kamarul et al. 2006
	–	FJ223864	Malaysia	Kamarul et al. 2006
H. (M.) leucospilota	JN207617	JN207541	Marshall Islands (Majuro)	Honey-Escandón et al. 2012
	KC405566	KY986424	Pangkor Island, Malaysia	Kamarudin and Rehan 2015
	KY986417	KY986423	Pangkor Island, Malaysia	Kamarudin and Rehan 2015
	KC405565	KY986422	Pangkor Island, Malaysia	Kamarudin and Rehan 2015
H. (T.) impatiens	MK477999	MK477995	Colombia (Pacific) (INV EQU4236)	This study
	JN207632	JN207526	Mexico (Pacific)	Honey-Escandón et al. 2012
H. (T.) aff. impatiens	–	FJ223857	Malaysia	Kamarudin et al. 2010
H. (T.) arenicola	JN207608	JN207556	Florida (USA)	Honey-Escandón et al. 2012
*Isostichopus fuscus*	MK477908	MK477869	Panama (Pacific) IfTa210	**This study**

## Results

### Order Holothuriida Miller, Kerr, Paulay, Reich, Wilson, Carvajal & Rouse, 2017

#### Family Holothuriidae Burmeister, 1837

**Genus *Holothuria* Linnaeus, 1767**

**Subgenus Mertensiothuria Deichmann, 1958**

##### Holothuria (Mertensiothuria) viridiaurantia sp. nov.

Taxon classificationAnimaliaHolothuriidaHolothuriidae

77F1DEDC-BA5E-5BD9-8111-10420E6A5C86

http://zoobank.org/3CF88C56-4A82-4758-B74A-395DF68C7F68

**Figure 2.**
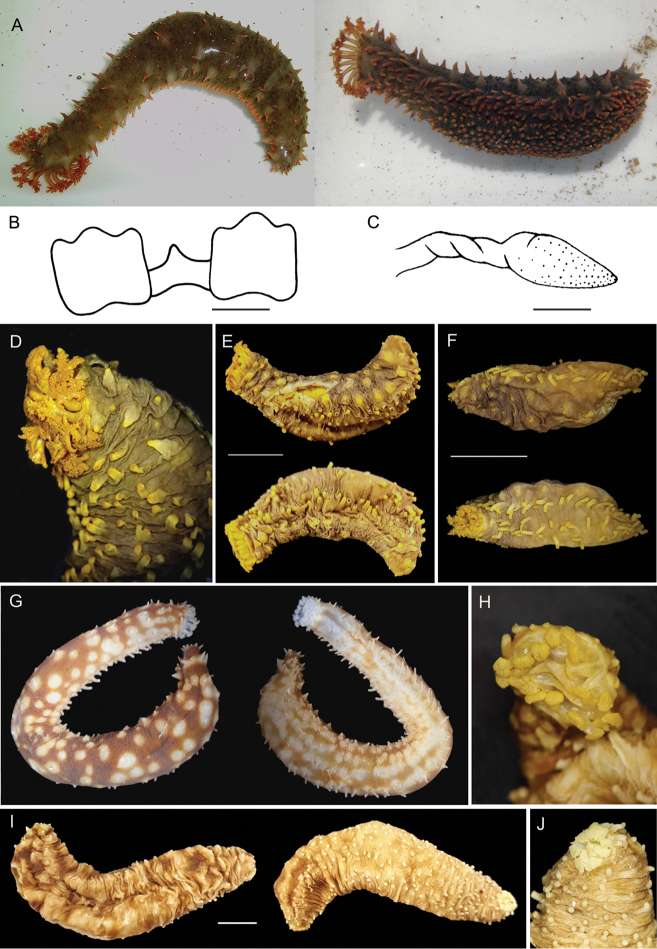
Type specimens of Holothuria (Mertensiothuria) viridiaurantia sp. nov. (**A–F**) and comparative material of Holothuria (Mertensiothuria) hilla (**G–J**). **A** Dorsal and ventral view of the alive holotype of H. (M.) viridiaurantia sp. nov. from Gulf of Cupica, Northern Chocó, Colombia (INV EQU4309, L = 70 mm) **B** calcareous ring and **C** stone canal and madreporite of the Holotype **D** detail of the preserved holotype tentacles **E** paratype from Cabo Marzo; Northern Chocó, Colombia (INV EQU4234, L = 35 mm) **F** smallest paratype from Gulf of Tribugá, Northern Chocó, Colombia (INV EQU4312, L = 25 mm) **G, H** alive specimen of H. (M.) hilla from Cabo Marzo, Northern Chocó, Colombia, and detail of tentacles in the preserved specimen (INV EQU4310, L = 100 mm) **I, J** preserved specimen of H. (M.) hilla, same locality as **G, H** and detail of tentacles (INV EQU4311, L = 65 mm). Scale bars: 2 mm (**B, C**); 1 cm (**E, F, I**).

**Figure 3.**
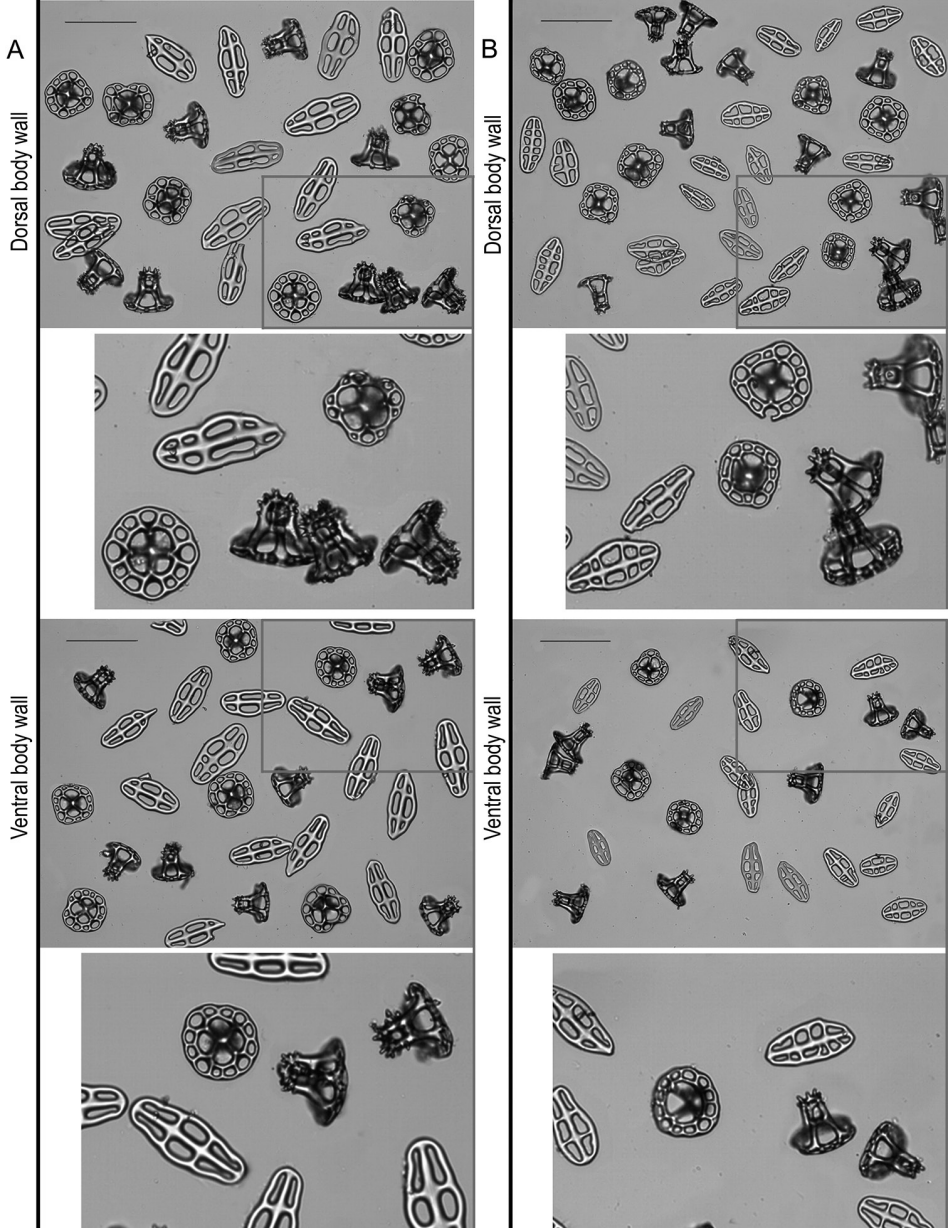
Ossicle comparison between Holothuria (Mertensiothuria) viridiaurantia sp. nov. and Holothuria (Mertensiothuria) hilla. **A** Holotype of H. (M.) viridiaurantia sp. nov. (INV EQU4309, L = 70 mm) **B**H. (M.) hilla (INV EQU4311, L = 65 mm); showing ossicle set from dorsal body wall (tables, buttons) and ventral body wall (tables, buttons); grey squares indicated in the images are presented enlarged below each image. Scale bar: 100 µm.

**Figure 4.**
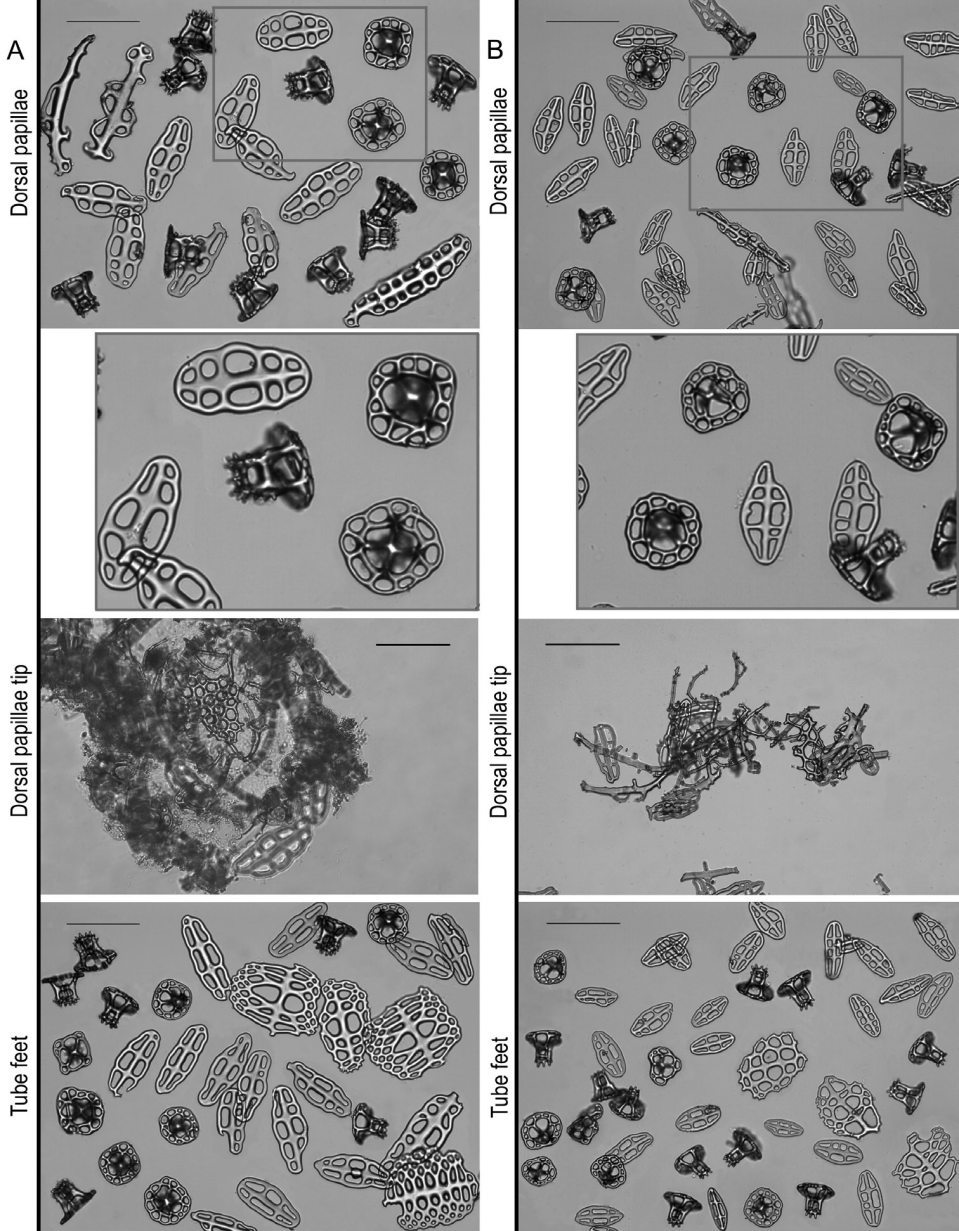
Ossicles comparison between Holothuria (Mertensiothuria) viridiaurantia sp. nov. and Holothuria (Mertensiothuria) hilla. **A** Holotype of H. (M.) viridiaurantia sp. nov.(INV EQU4309, L = 70 mm **B**H. (M.) hilla (INV EQU4311, L = 65 mm); showing ossicle set from dorsal papillae (tables, buttons, rods), dorsal papillae tip (showing the plate and small rods at the tip) and tube feet (tables, buttons and supporting plates); grey squares indicated in some images are presented enlarged below each image. Scale bar: 100 µm.

**Figure 5.**
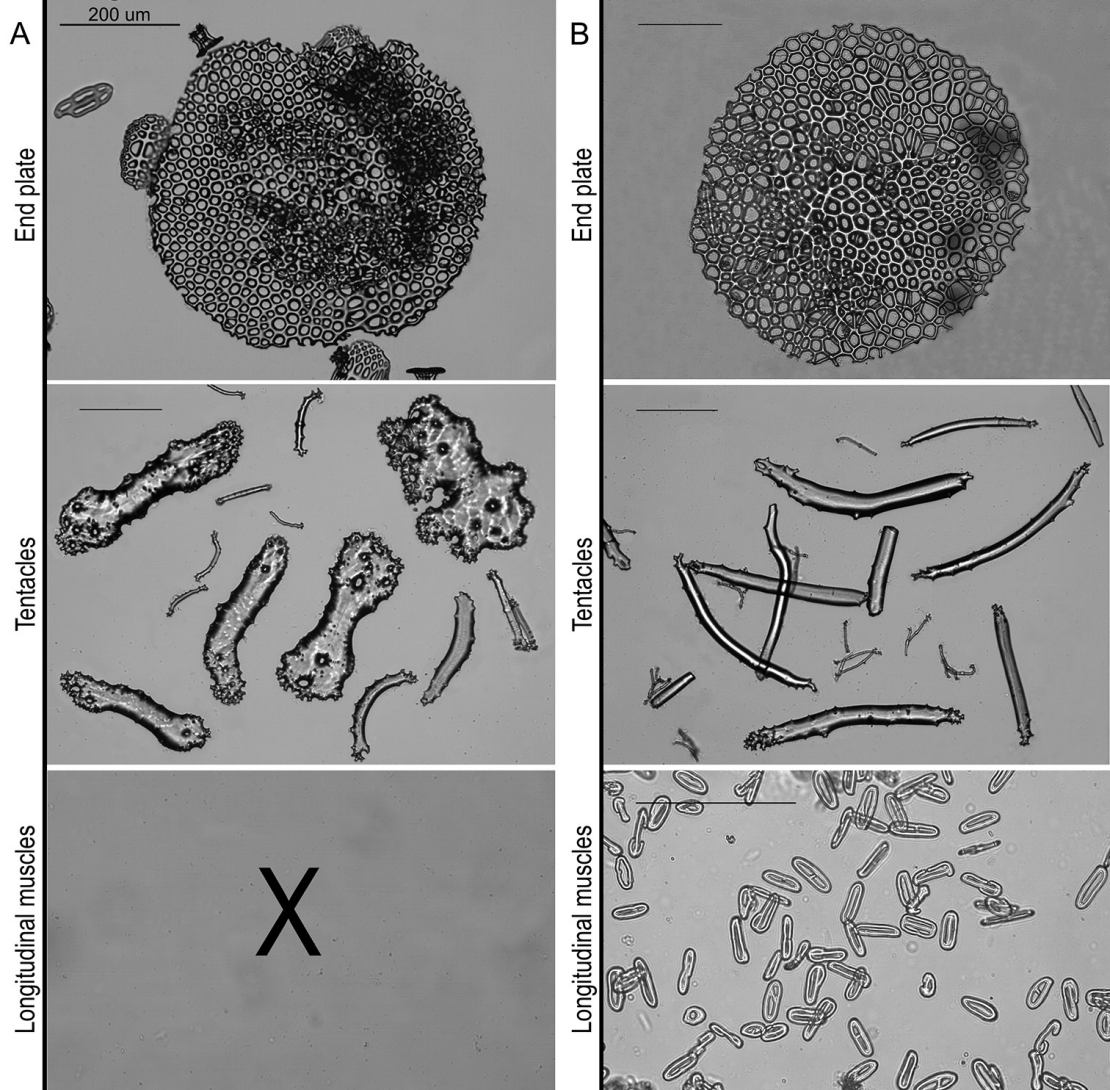
Ossicles comparison between Holothuria (Mertensiothuria) viridiaurantia sp. nov. and Holothuria (Mertensiothuria) hilla. **A** Holotype of H. (M.) viridiaurantia sp. nov. (INV EQU4309, L = 70 mm) **B**H. (M.) hilla (INV EQU4311, L = 65 mm); showing end plates from tube feet and ossicle set from tentacles (large and small rods) and longitudinal muscles (C's and O's ossicles). Scale bars: 100 µm (except **A** upper).

**Figure 6.**
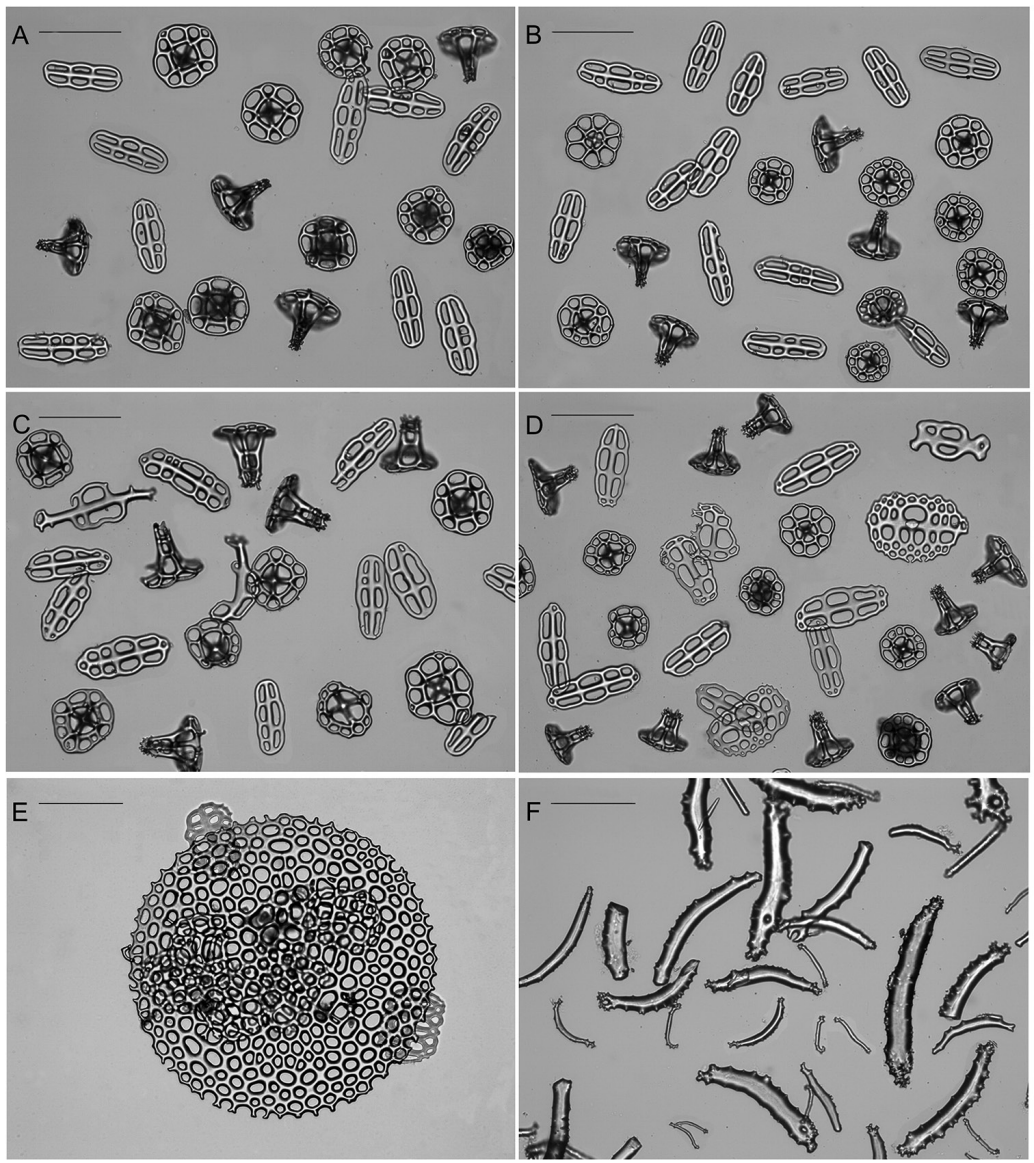
Ossicles of the juvenile paratype of Holothuria (Mertensiothuria) viridiaurantia sp. nov. (INV EQU4312, L = 25 mm). **A** Dorsal body wall (tables, buttons) **B** ventral body wall (tables, buttons) **C** dorsal papillae (tables, buttons, rods) **D** tube feet (tables, buttons, supporting plates) **E** tube feet (end plate) **F** tentacles (large and thick rods, small rods). Scale bars: 100 µm.

**Figure 7.**
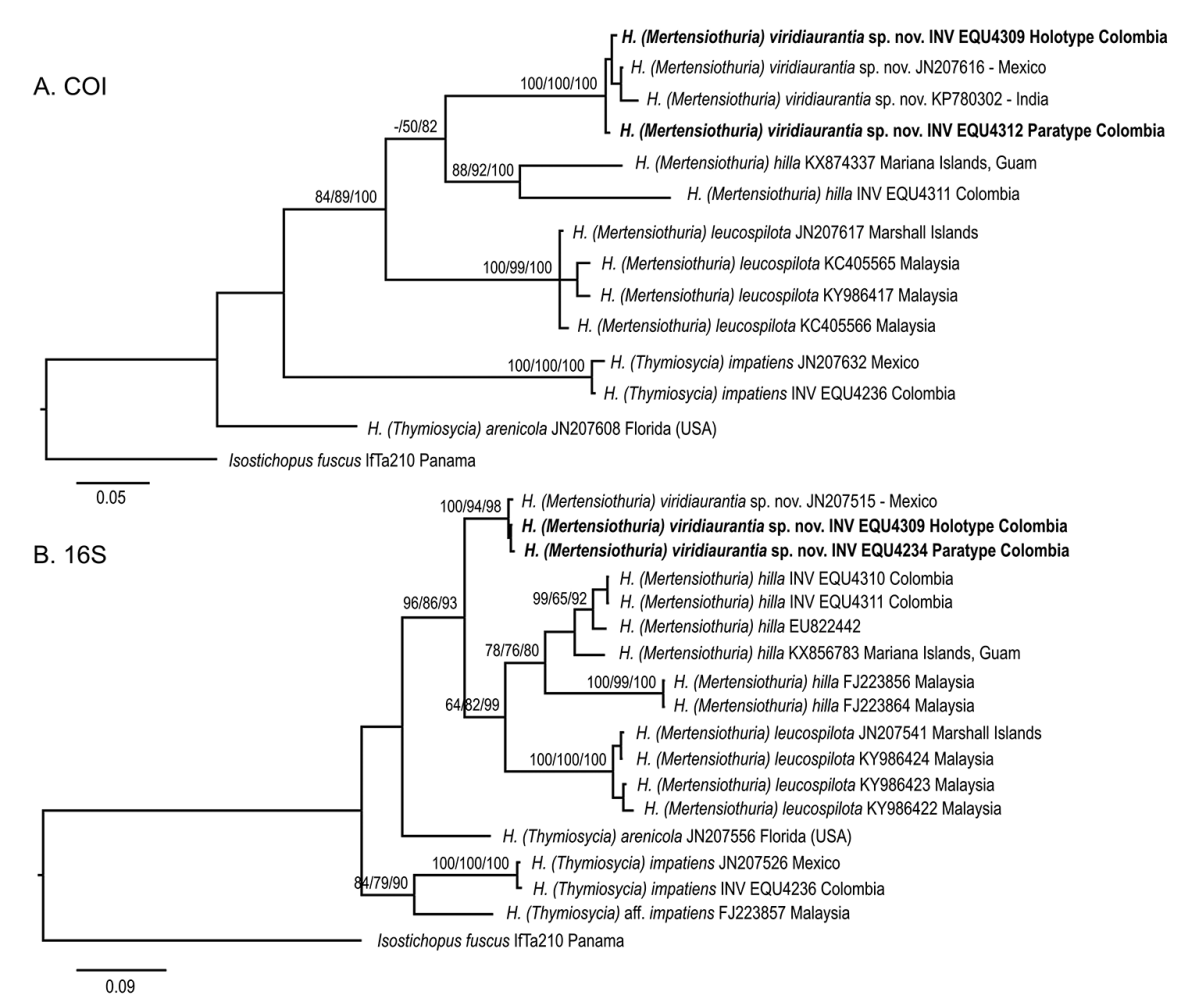
Bayesian inference tree of mitochondrial genes of the specimens analysed for the present study. **A**COI**B** 16S. The numbers on the nodes indicate Neighbour Joining (bootstrap %) / Maximum Likelihood (bootstrap %) / Bayesian posterior probability. Hyphen (-) indicates nodes not supported in some trees. Sequences from Colombia obtained in this study include the catalogue number at the MHNMC - INVEMAR (INV EQU); GenBank ascension number is included for the other sequences (see Table 1).

Figures 1 , 2 , 3 , 4 , 5 , 6 , 7 ; Tables 1 , 2 , 3

Holothuria (Mertensiothuria) hilla Solís-Marín et al. 2009: 110–111, fig. 30A–G; Santos-Beltrán and Salazar-Silva 2011: fig. 2A; Honey-Escandón et al. 2012; Sotelo-Casas et al. 2015: 3–4, figs 2E, 3(4–8), 4(4–6); Molina et al. 2015: fig. 3C.

###### Material examined.

***Holotype***: INV EQU4309, one specimen, total length 70 mm; collected in La Viuda rocky reef, Gulf of Cupica, northern Chocó, Colombia (6°37.9812'N, 77°29.985'W), by G. Borrero, 24Oct 2016; at 15 m depth under rocks and attached; GenBank nucleotide sequences COIMK477997 and 16S MK477991 (Fig. 2A–D). ***Paratype***: INV EQU4234, one specimen, total length 35 mm; collected in El Faro rocky reef, Cabo Marzo, northern Chocó, Colombia (6°49.4802'N, 77°41.3976'W), by M.J. Vanegas, 24 April 2016; at 13 m depth under rocks and attached; GenBank nucleotide sequences 16S MK477992 (Fig. 2E); ***Paratype***: INV EQU4312, one specimen, total length 25 mm; collected in Morromico rocky reef, Gulf of Tribugá, northern Chocó, Colombia (5°52.3194'N, 77°18.6426'W), by G. Borrero, 20 Oct 2016; at 10 m depth under rocks; GenBank nucleotide sequences COIMK477998 (Fig. 2F).

###### Comparative material

**Holothuria (Mertensiothuria) hilla**: INV EQU4245, one specimen, total length 75 mm; collected in Piedra de Rodrigo rocky reef, Cabo Marzo, northern Chocó, Colombia (6°47.0346'N, 77°41.6148'W), by M.J. Vanegas, 25 April 2016; at 19 m depth under rocks; INV EQU4310, four specimens, total length 70–100 mm; by G. Borrero, 26 Oct 2016; same locality, depth, and habitat as previous; GenBank nucleotide sequences 16S MK477993 (Fig. 2G, H); INV EQU4311, one specimen, total length 65 mm; by G. Borrero, 26 Oct 2016; same locality, depth, and habitat as previous; GenBank nucleotide sequences COIMK477996 and 16S MK477994 (Fig. 2I, J).

###### Diagnosis.

Olive-green background with white-orange dorsal papillae, tube feet and tentacles; buttons >75 µm in length; large tentacles with deep indentations; tentacle rods thick, rough and with some perforations; longitudinal muscles without ossicles.

###### Description.

External appearance: medium-sized species, holotype preserved specimen 70 mm long and 21 mm wide; body loaf like (length < 4× diameter) length/width ratio 2.3. Body shape of living ex situ specimen cylindrical in cross-section (Fig. 2A), tapering posteriorly and widening anteriorly, ending in a large crown of tentacles. Body wall soft and thin (2–3 mm thick). Anus terminal surrounded by small papillae. Mouth directed ventrally in live and preserved specimens, encircled by large papillae (Fig. 2A, D). Large peltate tentacles 20; ca. 5–6 mm total length, and 4–5 mm width shield; with deep indentions 2–3 mm. Few large, long and slender conical papillae scattered on the dorsal surface, although a vague arrangement into four rows is observed, two of them are lateral, where they are a little larger; smaller papillae scattered among the largest. Ventral tube feet cylindrical, large and thick, densely distributed throughout the ventral surface.

***Colour*.** Background of living specimens olive-green; base of the papillae is a light or whitish green that changes to orange from the middle to the ends, however, the tips of the papillae are whitish. Ventral surface similar to dorsum, with orange tube feet and white suckers; tentacles orange, same colour as papillae and tube feet (Fig. 2A). Dark brownish green in preserved specimens with papillae, tube feet, and tentacles a dark yellow (Fig. 2D–F).

***Internal anatomy.***

Square radial plates in the calcareous ring, 3 mm wide and 3 mm high, with three anterior rounded processes, and posterior margin with shallow rounded indentation; interradial plates slender, 1.5 mm high and 2.5 mm wide, pointed anterior margin and rounded posterior margin (Fig. 2B). One free stone canal, 4 mm long, and a helicoidally madreporite, 4 mm long (Fig. 2C). Tubular tentacle ampullae, 3–4 mm long and striped coloured. Tube-like polian vesicle, 17 mm long. Longitudinal muscles pair flat, thinner in the middle of each pair, irregularly wide, 3–4 mm wide each band, or 2–2 mm wide, attached, with narrow free edges. Gonads absent. Cuvierian organ present. Right respiratory tree extending to anterior end; left respiratory tree attached to the intestine until the middle of the body.

***Ossicles***: Dorsal and ventral body wall include similar tables and buttons, with dorsal tables taller and dorsal buttons larger than ventral (Table 2, Fig. 3A). Tables disc circular to quadrangular in outline; rim of the disc smooth; with four large central perforations and 7–12 smaller peripheral holes, arranged in one ring; spire with four pillars, single crossbeam, spiny crown with a small central hole (Fig. 3A). Dorsal tables 60–81 µm across disc (x- = 68 µm) and 43–54 µm height (x- = 49 µm); ventral tables 57–71 µm across disc (x- = 63 µm) and 40–49 µm height (x- = 44 µm). Buttons with smooth rim but irregular contour, usually with three pairs of holes, sometimes four pairs or three-four unpaired holes (Fig. 3A). Dorsal buttons 79–115 µm long (x- = 101 µm); ventral buttons 82–108 long (x- = 94 µm). Dorsal papillae with tables, buttons, button-like plates, rods and at the very tip one small plate and small rods (Table 2, Fig. 4A). Tables and buttons similar in shape and size to the ones in the body wall, although table’s spires are thicker and buttons are larger, up to 130 µm. Rods 167–203 µm long (x- = 187 µm) with distal or median perforations; small plates at the top of the papillae 99–134 µm (x- = 187 µm) and small rods 37–58 µm (x- = 48 µm). Ventral tube feet or pedicels with tables, buttons, plates, and end plates (Table 2, Figs 4A, 5A). Tables and buttons similar in shape and size to the ones in ventral body wall, although buttons are larger, up to 140 µm. Plates 105–133 µm long (x- = 117 µm) and 72–129 µm wide (x- = 116 µm); end plates 578–581 µm wide. Tentacles with large and small rods (Table 2; Fig. 5A); large rods are thick plate-like and very rough, usually with perforations at the extremities or along its length, 113–261 µm long and 33–150 µm width. Small rods thin and few spinose (58–107 µm). Longitudinal muscles without ossicles (Fig. 5A), as well as in the other internal organs, such as respiratory trees, tentacle ampullae, cloaca, and intestine.

**Table 2.** Comparison of ossicle size in Holothuria (Mertensiothuria) viridiaurantia sp. nov. holotype, juvenile paratype, and H. (M.) hilla of similar body size.

Characteristics	H. (M.) viridiaurantia sp. nov. Paratype INV EQU4312		H. (M.) viridiaurantia sp. nov. Holotype INV EQU4309		H. (M.) hilla INV EQU4311	
	L = 25 mm		L = 70 mm		L = 65 mm	
	Range (µm)	Average	Range (µm)	Average	Range (µm)	Average
**Dorsal body wall**						
Buttons length	88–119	99	79–115	101	47–70	61
Tables disc diameter	62–84	73	60–81	68	51–75	59
Tables height	56–68	62	43–54	49	35–45	41
Width spires	14–15	15	23–31	29	21–26	23
**Ventral body wall**						
Buttons length	78–104	90	82–108	94	50–73	61
Tables disc diameter	54–80	63	57–71	63	47–61	53
Tables height	45–56	51	40–49	44	34–44	39
Width spires	9–17	14	22–29	25	16–20	18
**Dorsal papillae**						
Buttons, Buttons-like plates length	80–111	99	106–130	117	64–91	79
Tables disc diameter	51–95	74	60–82	71	48–66	58
Tables height	58–71	64	42–62	52	43–47	45
Width spires	19–28	25	30–42	36	20–25	22
Rods	97–168	143	167–203	187	102–152	129
Plates	–	-	99–134	118	104–107	106
Small Rods	–	-	37–58	48	43–67	57
**Ventral tube feet**						
Buttons, Buttons-like plates length	93–116	105	91–140	110	52–84	70
Tables disc diameter	54–75	63	53–73	61	41–57	51
Tables height	46–54	50	41–51	45	34–43	38
Width spires	15–23	18	23–31	28	17–24	21
Plates length	66–122	96	105–133	117	76–127	96
Plates width	46–83	65	72–129	116	68 –99	84
End Plates	391–401	396	578–581	580	570–584	577
**Tentacles**						
Large Rods Length	132–259	197	185–261	221	166–267	227
Large Rods Width	21–39	29	33–150	75	10–27	18
Small Rods Length	37–91	56	58–107	74	37–65	47
**Longitudinal muscle**						
C-O shape ossicles length	NA	NA	NA	NA	13–33	24

***Paratypes***: Juveniles, 35 and 25 mm long, 12 and 8 mm wide respectively (Fig. 2E, F). External morphology different to the holotype, which is much larger at 70 mm long. Small dorsal papillae in the four main rows, as described for the holotype; and three rows of tube feet, two lateral and one in the middle of the ventral side which includes two irregular lines of pedicels (Fig. 2F). Dorsal and ventral body wall buttons are smaller in the juvenile, although there is not a considerable difference in size; however, in shape they are more rounded at the extremities and frequently present more than three pairs of holes (Table 2, Fig. 6A, B). Tables showed more changes during growth in comparison with buttons: the tables spire are taller and narrower, pointed-like without cross beam clearly noted, with few spines around the top; and the tables disc diameter is larger, with peripheral holes less in number and larger in size in the juvenile (Table 2, Fig. 6A, B). Dorsal papillae and tube feet present similar pattern of change during growth in buttons and tables when comparing the juvenile with the holotype; however, tables in dorsal papillae and tube feet in the juveniles are less pointed-like and one cross beam is clearly noted in most of the tables in comparison with those from the dorsal and ventral body wall (Fig. 6C, D). In addition, rods in dorsal papillae are smaller in size; it was not possible to observe the small plates and rods at the very top of the papillae. Supporting plates and end plates in the tube feet are also smaller in the juvenile (Table 2, Fig. 6D, E). Tentacle rods are not well developed in the paratype, being almost similar in length but less thick than those of the holotype, however, they are thicker than those in the H. (M.) hilla individual of 65 mm in length (Table 2, Figs 5A, B; 6F).

###### Etymology.

From the Latin *viridis* (green) and *aurantius* (orange-coloured), referring to the living colour with olive-green background and orange-white papillae, tube feet, and tentacles (feminine).

###### Distribution.

Holothuria (Mertensiothuria) viridiaurantia sp. nov. is known and confirmed along the Eastern Pacific from Mexico (as Holothuria (Mertensiothuria) hilla, Solís-Marín et al. 2009; Santos-Beltrán and Salazar-Silva 2011; Honey-Escandón et al. 2012; Sotelo-Casas et al. 2015), Panama (as H. (M.) hilla, Molina et al. 2015) and Colombia (present study) (Fig. 1). However, a GenBank sequence of one specimen from Kerala coast, India (Accession number KP780302.1) suggests that the new species could have a wider geographical distribution across the Indian Ocean and the Pacific Ocean to the Central and Tropical Eastern Pacific, like H. (M.) hilla (Fig. 1C). However, it was not possible to review the specimen belonging to the sequence, so colouration and morphological characteristics described in the present paper should be reviewed and confirmed. Notably, images of green-coloured H. (M.) hilla from the Philippines are presented by Dolorosa et al. (2017; Fig. 2J).

###### Habitat.

Holothuria (Mertensiothuria) viridiaurantia sp. nov. is associated with rocky bottoms from the intertidal to 15 m depth (Molina et al. 2015; present study). Specimens collected in Colombia were found attached under medium rocks, differing from H. (M.) hilla which were found under rocks but on a sandy substrate instead of a rocky substrate. Holothuria (M.) viridiaurantia sp. nov. was collected in three different rocky reefs in the northern Chocó, whereas H. (M.) hilla was found only in one, during both collection trips in 2016. Several specimens of H. (M.) hilla were found regenerating new anterior ends, this was not observed in specimens of the new species.

###### Conservation status.

As the specimens of Holothuria (Mertensiothuria) viridiaurantia sp. nov. were previously assigned to H. (M.) hilla, the conservation status of this species must be considered. Currently H. (M.) hilla is included in the IUCN Red List in the category of Least Concern, and in addition, it is classified as a low-value species (about USD3 kg^-1^ dried in the Philippines) among commercially important sea cucumbers of the world (Purcell et al. 2012). There is no fishery reports of H. (M.) hilla in the Eastern Pacific Ocean, however, H. (M.) hilla is fished commercially in the Philippines, Indonesia and Madagascar, that could include the new species considering the potential wider distribution of H. (M.) viridiaurantia sp. nov.

###### Remarks.

The new species was previously assigned to Holothuria (Mertensiothuria) hilla (Solís-Marín et al. 2009; Santos-Beltrán and Salazar-Silva 2011; Honey-Escandón et al. 2012; Molina et al. 2015; Sotelo-Casas et al. 2015), however there is no mention of the distinct and striking colouration of the specimens reported in those papers in comparison with H. (M.) hilla. Perhaps the identification of this species was based on the similar external appearance (shape of the body and papillae) and apparent similar ossicles at first sight; without regard to the colouration, which has been traditionally considered to be intra-specific variability in echinoderms. However, recent research demonstrates that it can be a diagnostic characteristic, for example in the species complex H. (T.) impatiens (Michonneau 2015); this subject requires careful and exhaustive study, especially the purpose of colouration in sea cucumbers (Clark 1922; Michonneau 2015). In this study, a detailed revision of specimens from the new species and H. (M.) hilla, showed not only the colouration as a diagnostic feature, but also the size and shape of the tentacles, which are larger and with deeper indentations in the new species (Fig. 2). In reference to the ossicles, although similar in shape at first sight, a detailed revision showed several diagnostic characteristics: 1) differences in the size of the complete ossicle sets from the dorsal and ventral body wall, dorsal papillae and tube feet; specifically, the tables are taller and thicker with wider discs and the buttons are larger in the new species, in both juvenile and large specimens (Table 2; Figs 3–6); size of the buttons is the most diagnostic trait for the species; 2) the size and shape of the tentacle rods, being wide (plate-like), thick and very rough, and with some perforations in the new species compared to slender rods in H. (M.) hilla (Table 2; Figs 5A, B, 6F); 3) longitudinal muscle ossicles are absent in the new species, contrary to H. (M.) hilla (Table 2; Fig. 5A, B). In general, the morphological structures of the new species are thicker and stronger than those of H. (M.) hilla, which is a more delicate species. Among the morphological characteristics of the new species, the absence of ossicles in the longitudinal muscles, larger size of the perforated plates of the tube feet, and size and shape of the tentacle ossicles, match those considered by Samyn and Massin (2003) for excluding *Holothuria arenacava* and *Holothuria platei* from *Mertensiothuria*. However, the decision for including the new species in this subgenus was made based on the mtDNA evidence.

###### Molecular characteristics.

We obtained COI and 16S sequence data from three specimens of Holothuria (Mertensiothuria) viridiaurantia sp. nov. and two of Holothuria (Mertensiothuria) hilla from the rocky reef in northern Chocó, Colombia. Specimens of H. (M.) viridiaurantia sp. nov. from Colombia (type specimens) were recovered in a well-supported clade, separated from H. (M.) hilla for both, COI and 16S genes (Fig. 7). Two sequences, derived from one specimen from Mexico (GenBank Accession No. JN207616–COI and JN207515–16S) and one from India (KP780302–COI), were recovered in the same clade as type specimens from Colombia. However, different tree topologies for COI and 16S sequence data were recovered. For COIH. (M.) viridiaurantia sp. nov. appears sister to H. (M.) hilla, with H. (M.) leucospilota positioned sister to them (Fig. 7A). However, for 16S H. (M.) hilla and H. (M.) leucospilota appeared as sister species with H. (M.) viridiaurantia sp. nov. as sister clade (Fig. 7B). Species from Thymiosycia subgenus appear separated from Mertensiothuria subgenus for both genes and all tree reconstruction methods (Fig. 7A, B). Evidence for species status of H. (M.) viridiaurantia sp. nov. comes from the COI and 16S genetic distances. Inter-specific distances between the two previously recognised *Mertensiothuria* species included in the analysis is 17.7% for COI and 13.9% for 16S; and distances between the new species and them are 16.7 and 15.6% for COI and 12.5 and 11.8% for 16S; inter-specific distances among species of *Mertensiothuria* and *Thymiosycia* showed larger values (Table 3). In addition, intra-specific distances for H. (M.) viridiaurantia sp. nov. were 1.31% for COI and 0.5% for 16S, the lowest values in all the species analysed. Intra-specific distances for H. (M.) hilla (13.8% for COI and 7.9% for 16S) could be showing a species complex, similar to what was described by Michonneau (2015) for H. (T.) impatiens, which is also recovered here with 8.8% for 16S, including one specimen identified as H. (T.) aff. impatiens (Table 3). Lower intraspecific distance for COI (0.9%) for H. (T.) impatiens is explained because the sequence for COI was not available for this specimen. There is, therefore, strong molecular evidence that H. (M.) viridiaurantia sp. nov. is an undescribed species different from H. (M.) hilla, a finding also supported by the morphological characteristics described previously.

**Table 3.** Kimura 2 parameter distances (%) within specimens of Holothuria (Mertensiothuria) viridiaurantia sp. nov. and between the *Holothuria* species included in the analysis. COI distances are below diagonal and 16S distances above. The numbers in bold lettering along the diagonal represent average within species distances for COI and 16S (COI / 16S).

Species		1	2	3	4	5	6
**1**	H. (M.) viridiaurantia sp.nov.	**1.3 / 0.5**	12.5	11.8	15.7	13.5	32.6
**2**	H. (M.) hilla	16.7	**13.8 / 7.9**	13.9	19.4	16.3	33.9
**3**	H. (M.) leucospilota	15.6	17.7	**1.9 / 1.6**	19.4	17.2	34.8
**4**	H. (T.) impatiens	21.3	21.5	21.8	**0.9/ 8.8**	15.5	33.2
**5**	H. (T.) arenicola	18.0	19.9	17.6	19.4	**nc / nc**	34.3
**6**	*Isostichopus fuscus*	24.4	22.5	24.0	24.5	20.7	**nc / nc**

## Supplementary Material

XML Treatment for Holothuria (Mertensiothuria) viridiaurantia
